# Migraine and Happiness

**DOI:** 10.1089/whr.2021.0122

**Published:** 2022-02-02

**Authors:** Heiko Pohl, Anne-Catherine Streit, Maria S. Neumeier, Gabriele S. Merki-Feld, Willibald Ruch, Andreas R. Gantenbein

**Affiliations:** ^1^ Department of Neurology, University Hospital Zurich, Zurich, Switzerland.; ^2^ Swiss Epilepsy Clinic, Klinik Lengg, Zurich, Switzerland.; ^3^ Department of Reproductive Endocrinology, University Hospital Zurich, Zurich, Switzerland.; ^4^ Department of Psychology, Section on Personality Psychology and Assessment, University of Zurich.; ^5^ Zurzach Care, Bad Zurzach, Switzerland.

**Keywords:** satisfaction, coping, happy, satisfaction with life scale, opponent-process theory, hedonic habituation

## Abstract

***Objective:*** To investigate the association between happiness and migraine.

***Background:*** Contemporary operationalizations of happiness include the prevailing positive over negative affect and the satisfaction with life. Generally, extreme events and circumstances influence happiness only temporarily. However, how does periodic cycling between being relatively healthy and relatively disabled—as in migraineurs—affect happiness? Migraine is a primary headache disorder, in which headache attacks intermittently interfere with normal living and cause a significant personal, societal, and potentially irreversible disease burden.

***Methods:*** In this cross-sectional observational study, migraineurs completed the satisfaction with life scale (SWLS), the Patient Health Questionnaire, and the Generalized Anxiety Disorder scale and reported their headache frequency as well as recent changes in that frequency. Furthermore, participants answered a free text question on how to remain happy despite migraine attacks. We built a regression model with the SWLS score as the dependent variable.

***Results:*** Seventy participants completed the questionnaire. The regression model revealed that happiness increases with headache days, and subsequent analysis showed a U-shaped relationship between headache frequency and happiness. The participants' advice on remaining happy focused on upvaluing the pain-free time or relieving the attacks themselves. The latter was increasingly common with longer disease durations.

***Conclusions:*** Both high and low headache frequencies facilitate adaptation to the disorder, while intermediate frequencies resulted in lower life satisfaction. The nonlinear relationship between happiness and headache days may be due to “hedonic habituation” and implies that headache calendars do not necessarily correctly reflect patients' difficulty to feel well despite the disorder. Many patients advised other migraineurs to increase happiness by enjoying pain-free time. However, with increasing disease duration, patients' recommendations focused on coping with attacks.

## Introduction

Throughout the centuries, different schools of thought conceived differing understandings of happiness, differing degrees to which individuals can reach it themselves, and different means to obtain it.^1^

According to Herodot, Solon understood happiness as a posthumous evaluation of life's good and bad aspects. He assumed that obtaining happiness is beyond humans' control and depends entirely on gods' will and outer circumstances—such as the absence of plague, famine, and hostility. The etymological relationship between “happiness” and “chance” encountered widely across linguistic and cultural borders witnesses this concept's popularity.^1^

Later, Aristotle and Plato tentatively considered humans as potentially having *some* influence on obtaining happiness. Stoics aimed to reach it *despite* adverse conditions, and Jesus taught to attain it *because of* them (Matthew 5:3–12).^1^

A shift in understanding happiness occurred slowly when philosophers gradually came to view happiness not as an objective state but as subjective. As a result, contemporary research distinguishes different operationalizations of the term, including the prevailing positive over negative affect and the satisfaction with life.^2,3^ The latter alludes to the ancient understanding of the term as it implies drawing a balance of the life lived so far.

Although outer circumstances impact happiness in this modern understanding, they usually do so only transiently—probably because humans quickly accustom themselves to their fate.^4^ However, what happens if ever-changing circumstances impede adaptation? More precisely, how does periodic and often unpredictable cycling between being relatively healthy and relatively disabled—as in migraineurs—affect happiness?

Migraine is a primary headache disorder that affects about 12% of the Caucasian population, with a higher prevalence in women than in men.^5^ Attacks last between 4 and 72 hours and—strongly interfering with normal living—cause a significant personal, societal, and potentially irreversible disease burden.^6–9^ Even though women and men between 35 and 45 are most often affected, headache attacks may occur at any time in life.^5^

Intolerance to sound and light, difficulties to focus, the need to lay down, the aversion to food, nausea, and vomiting, together with intense pain, characterize migraine attacks.^9^ This temporary debilitation forces affected persons to postpone meeting obligations or to fail them entirely. Many patients believe that their migraine made them less successful in their careers.^6^ Hence, it suggests itself that migraine interferes with happiness.

Previous studies underlined that most people pursue and attribute great importance to happiness.^10^ Therefore and because primary headache disorders can be managed, not cured, it is crucial not just to reduce the number of attacks but attempt to help patients thrive and live happily despite the disorder.^11^ However, it is unknown whether migraine influences happiness.

This study aims to investigate for the first time the association between happiness and migraine.

## Methods

### Study design

We conducted this cross-sectional observational study from October 2019 to June 2021. Initially, we recruited all participants from our tertiary care headache outpatient clinic. We had diagnosed migraine according to the operational definition published in the third edition of the International Headache Society in all of them.^9^ The diagnostic criteria stipulate that the headache appears in attacks with a duration of 4–72 hours, and moderate or severe pain intensity that increases with physical activity. The pain usually has a pulsating quality and is often unilateral. Nausea, vomiting, and/or photophobia and phonophobia accompany the attacks.

However, as the participation rate remained low, from September 2020, we also sent invitations through mailing lists inviting persons not under our treatment.

Adults with a migraine diagnosis were eligible to participate. Upon inviting participants through mailing lists addressing mainly students of the University of Zurich, we asked them to respond to a screening question that assessed one inclusion criterion (“Has a physician ever diagnosed you with a migraine?”). Before that, we had only invited patients diagnosed as migraineurs in our clinic, so the screening question had been dispensable.

Having provided their informed consent, all participants anonymously completed an online questionnaire that comprised the satisfaction-with-life-scale (SWLS) a five item questionnaire measuring happiness,^2^ which was developed by Diener and coworkers in 1985.

Moreover, participants also completed the Patient Health Questionnaire (PHQ-8),^12^ and the Generalized Anxiety Disorder scale (GAD-7). Moreover, participants reported their number of monthly migraine days (MMD) and monthly headache days, their average level of functioning during a migraine attack (defined as the proportion of planned activities actually executed during the headache attack^13^), the average duration of an attack (measured in hours and days), and the disease duration (<1 year, 1–2 years, 3–5 years, 6 − 10 years, >10 years).

We had asked participants to distinguish between migraine and headache days to remind them that not every headache day is necessarily a migraine day. Moreover, we also asked whether migraine frequency had changed during the last 3 months; answer options were “there was a strong increase,” “there was a small increase,” “there was no change,” “there was a small decrease,” and “there was a strong decrease.” Furthermore, we assessed sex and age. Finally, the last item of the questionnaire comprised a free text question asking participants to advise other migraineurs on how to live happily despite migraine attacks. We specified in each question whether it addressed migraine attacks or referred to headache attacks in general.

The research project did not fall under the human research act, as anonymous data were collected; accordingly, the Ethics Committee Zurich granted a waiver (REQ-2019-00788). The available data determine the sample size.

### Data analysis

We report categorical variables as frequencies and continuous variables as median with the 25th and the 75th percentiles put into brackets. Using the information on attack duration and level of functioning during an attack, we calculated the time lost due to an attack (TLA) multiplying the level of functioning (see above) with the duration of the attack.^13^

We interpreted the score of the SWLS as published^14^: Participants who scored below 10 points were “extremely dissatisfied,” those who scored between 10 and 14 points were “dissatisfied,” and between 15 and 19 points were “slightly dissatisfied.” Patients with 20 points were “neutral.” Participants scoring between 21 and 25 points were “slightly satisfied,” those scoring between 26 and 30 points were “satisfied,” and those scoring more than 30 points were “extremely satisfied.”

Spearman's Rho assessed the correlation between ordinal and continuous variables. The Mann-Whitney U-Test allowed assessing the influence of dichotomous variables on an ordinal scaled or continuous variable.

Furthermore, to estimate the influence of headache days on happiness, we built an ordinal regression model with the total score of the SWLS as the dependent variable. Independent variables were the number of monthly headache days, disease duration, and recent changes in migraine frequency, as well as age and sex. We also included the PHQ-8 and GAD-7 as covariates to control any confounding influence of comorbid anxiety and depression. In addition, we used the test of parallel lines and the Pearson Goodness-of-Fit Test and calculated Pseudo R-square Nagelkerke.

Finally, we analyzed the answers to the free text question investigating commonalities.

The term “not reported” (n.r.) indicates missing data. We used IBM SPSS version 25 for the statistical analysis and set the significance level at 0.05.

### Data availability

The data collected for this study are available from the corresponding author upon reasonable request.

## Results

### Quantitative analysis

Table 1 provides an overview of the demographic characteristics of the sample. Seventy participants met the inclusion criterion and filled in the questionnaire. Their median age was 28.5 years (22, 44 years); 62 were female (62/70, 88.6%). Most participants were either working (34/70, 48.6%) or studying (30/70, 42.9%). Two were unemployed (2/70, 2.9%), two were homemakers (2/70, 2.9%), and two were retired (2/70, 2.9%). More than half of the participants were single (38/70, 54.3%). Some were in a relationship (16/70, 22.9%) or married (11/70, 15.7%). Only one participant lived separated from their spouse (1/70, 1.4%), and two were divorced (4/70, 5.7%).

**Table 1.** Results of the Ordinal Regression Model with 63 Participants

Variable	Odds ratio	95% confidence interval	*p*
Change in MMD (strong increase)	1.538	0.308–7.683	0.600
Change in MMD (slight increase)	3.805	0.873–16.575	0.075
Change in MMD (slight decrease)	0.648	0.174–2.416	0.518
Change in MMD (strong decrease)	0.280	0.063–1.247	0.095
Change in MMD (no change)^*^			
Age	0.990	0.954–1.028	0.598
Disease duration	1.169	0.736–1.857	0.507
GAD-7	0.882	0.761–1.022	0.095
Monthly headache days	1.102	1.017–1.194	0.018
PHQ8	0.855	0.733–0.997	0.045
Sex (female)	0.451	0.095–2.152	0.318
Sex (male)^*^			

The score of the SWLS score was the dependent variable; the independent variables are listed below. When analyzing categorical variables, one value was chosen as the reference value and marked with an asterisk. An odds ratio >1 suggests that increasing values of this variable are associated with an increased score in the SWLS, whereas a ratio <1 suggests that it is associated with a decrease. GAD-7, Generalized Anxiety Disorder 7; MMD, monthly migraine days; PHQ8, patient health questionnaire 8; SWLS, satisfaction-with-life-scale.

The median number of MMD was 4 days (2, 9 days; 1 n.r.), the number of monthly headache days was 8 days (5, 12.5 days), and the number of monthly medication days was 5 days (3, 9 days). Twenty-three participants (23/70; 32.9%) took migraine prophylaxis.

During the last 3 months, no change in migraine frequency had occurred in 27 participants (27/70, 38.6%). Nine recalled a strong (9/70; 12.9%) and 18 a slight decrease (18/70; 25.7%); 7 recalled a strong (7/70, 10.0%) and 9 a slight increase (9/70; 12.9%) in migraine days.

The median level of disability during an attack was 70% (50, 90%; 1 n.r.); participants reported a median TLA of 11.2 hours (3.7, 24.0 hours; 4 n.r.) for untreated and 3 hours (1.4, 5.2 hours; 5 n.r.) for successfully treated attacks.

Disease duration was <1 year in one participant (1/70, 1.4%), 1–2 years in nine participants (9/70, 12.9%), 3–5 years in 19 participants (19/70, 27.1%), 6–10 years in 11 participants (11/70, 15.7%), and >10 years in 30 participants (30/70, 42.9%).

Sixty-five participants completed the SWLS and scored a median of 25 points (19.5, 28 points). Based on their answers, 6 were “very satisfied” (6/65, 9.2%), 22 were “satisfied” (22/65, 33.8%), 19 were “slightly satisfied” (19/65, 29.2%), 2 were “neutral” (2/65, 3.1%), 12 were “slightly dissatisfied” (12/65, 18.5%), 3 were dissatisfied (3/65, 4.6%), and 1 was “extremely dissatisfied” (1/65, 1.5%) with their lives. Overall, a majority was satisfied at least to some degree (47/65, 72.3%).^14^

The SWLS scores did not correlate with disease duration (*r* = 0.124, *p* = 0.326; 5 n.r.), nor with MMD (*r* = −0.034, *p* = 0.792, 6 n.r.), nor with monthly headache days (*r* = −0.049, *p* = 0.849, 5 n.r.), and nor with the level of disability during migraine attacks (*r* = 0.123, *p* = 0.334, 5 n.r.). Changes in MMD during the last 3 months did not influence this score (*p* = 0.292; 5 n.r.).

**FIG. 1.**
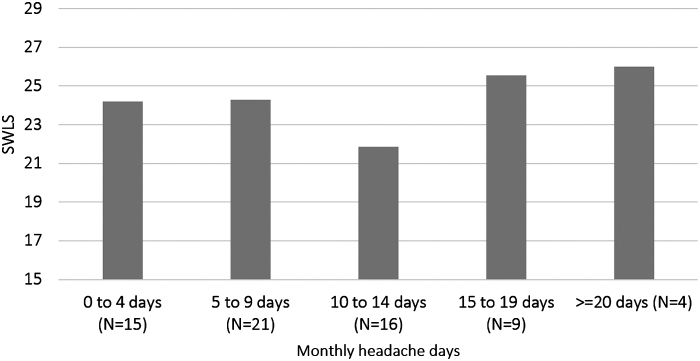
Relationship between monthly headache days and the average scores of the SWLS. SWLS, Satisfaction-With-Life-Scale.

Figure 1 depicts the relationship between the monthly headache days and average scores in the SWLS.

The median score of the PHQ-8 was 8 points (4, 10 points; 7 n.r.); 18 (18/63; 28.6%) scored 10 or more points and were therefore likely to suffer from a depressive disorder.^12^

The median score of the GAD-7 was 5 points (2.75, 9 points; 4 n.r.); 13 (13/66; 18.6%) scored 10 or more points and therefore had moderate to severe anxiety symptom levels.^15^

The data of 63 patients entered the regression model. Table 1 lists the results. Neither disease duration nor changes in MMD had an impact on happiness. However, the number of headache days had a statistically significant impact, with higher values associated with higher scores in the SWLS.

The test of parallel lines indicated that the proportional odds assumption was satisfied (*p* = 0.911). Likewise, the Pearson Goodness-of-Fit Test indicated a good fit (*p* = 0.636). Pseudo R-square Nagelkerke was 0.373.

### Qualitative analysis

Two-thirds of the participants (47/70, 67.1%) answered the free text question, totaling 95 pieces of advice. Of these, less than half (42/95, 44.2%) focused on the “good times” (*i.e.*, when pain-free) and often recommended appreciating and enjoying them. Some also suggested working out, going for walks, or meditating, and, more generally, increasing the “good times” (*e.g.*, by finding a good and meaningful job, taking time to relax, reducing stress, and opting for a balanced diet).

By contrast, a majority (53/95, 55.8%) of the recommendations focused on the “bad times” (*i.e.*, attacks) and, generally, fell into the categories “acceptance/mindset,” “acute treatment,” and “finding help/prevention.” Many pieces of advice falling into the first category recommended accepting or ignoring both the migraine and the associated disability—or at least not overthinking about them. Furthermore, they advocated being optimistic about better times to come and emphasized that all attacks eventually end.

Suggestions in the second category (“acute treatment”) advocated taking acute medication and looking for alternative forms of relief (*e.g.*, a towel soaked with cold water wrapped around the head, drinking a warm tea, breathing fresh air, and going for a walk).

In the last category (“finding help/prevention”), some participants recommended looking for and avoiding potential triggers, talking with others about their migraines, trying psychological treatment options, and consulting neurologists specializing in headache disorders. Notably, nobody mentioned pharmacological preventive treatment approaches.

Participants who had answered the free text question were not more or less happy than those who had not (*p* = 0.340). Moreover, those whose advice had focused solely on the “good times” (14/42, 33.3%; five participants' advice fit into both categories) did not score higher in the SWLS than those who had focused on the “bad times” (the median score was 25 in both groups, *p* = 0.847). Besides, the number of MMD did not differ significantly between the groups (*p* = 0.128). However, patients with longer disease durations focused more often on the “bad times” (*p* = 0.041).

## Discussion

In this study, we investigated the influence of migraine attacks on happiness. The main results were that most participants were at least to some degree satisfied with their lives and that the probability of a higher score in the SWLS (“happiness”) increases when the number of monthly headache days increases.

The association of headache days with higher scores of the SWLS was unexpected. We had anticipated that the constraints imposed by migraine would lead to reduced satisfaction with life. Notably, an increase in MMD during the last 3 months was also associated with higher life satisfaction (Table 1). Even though that finding did not reach statistical significance, it pointed in the same direction, suggesting that the association of happiness and migraine is indeed counterintuitive.

The univariate analysis had not detected any association of happiness and headache days—only the regression analysis did −, suggesting that other variables might sometimes obscure this effect. The covariate most likely to reduce happiness is depression (Table 1) because depressive symptoms correlate with positive and negative affect,^16^ which correlate with the score of the SWLS.^17^

Figure 1 suggests a slightly “U shaped” relationship between SWLS and headache days. Consequently, it is unlikely that headache days make anyone happy. Instead, an intermediate migraine frequency makes more, and a very high number of attacks makes less unhappy than lower numbers. We hypothesize that Solomon's Opponent-Process Theory may be applicable in this context.^18^

Assuming that migraine is a stimulus with negative valence, we would expect a heightened affect to follow the attack as an opponent process. Accordingly, some patients report “feeling high” after an attack,^19^ and consequently, a higher number of MMD might result in more and long-lasting happiness—as in our sample. However, not all patients experience such postdrome suggesting that this hypothesis is not generally correct.

Conversely, migraine attacks may be part of an opponent process themselves. Many patients report that their attacks do not occur randomly, but in response to certain stimuli experienced as stressful or exhausting.^20^ Consequently, migraine attacks may be part of a counterreaction associated with reduced stress and reduced energy consumption.

According to Solomon, when a threshold stimulus frequency is reached (the “critical decay duration”), both intensity and duration of the opponent process increase. This mechanism may explain the reduced happiness in patients with an intermediate number of migraine days (Fig. 1).^18^ When the stimulus frequency increases further, a “hedonic habituation” comes into play. This term refers to the reduced affective reaction to stimuli repeated in short intervals compared with stimuli repeated in longer intervals or presented only once.^18^ This mechanism may explain the increase in the scores of the SWLS in very high numbers of MMD.

The latter interpretation of migraine attacks as opponent processes requires that patients with no migraine attacks, on average, score higher in the SWLS than patients with more MMD. However, as we did not include a control group and the number of migraineurs with zero attacks was low, we cannot test that hypothesis. In addition, longitudinal studies of patients transitioning from frequent migraine days to daily pain and from a low number of migraine days to no migraine days may be necessary to prove that the opponent-process theory explains the affective reaction to migraine attacks.

Notably, the nonlinear relationship between SWLS and headache days (Fig. 1) implies that counting headache or migraine days does not necessarily correctly reflect patients' difficulty to thrive and to feel well despite the disorder. Patients with an intermediate number of headache days may suffer emotionally more than those with daily headaches do.

It is also important to note that most participants in this study were rather young and often had a relatively low number of migraine and headache days. Moreover, many were well integrated into the job market, suggesting well-functioning coping strategies. Consequently, our study might not capture the full effect of a chronic migraine disorder on happiness. Thus, we encourage further studies among patients with chronic migraine.

While—to our knowledge—no study has investigated life satisfaction in migraineurs, one study chose a similar approach to ours. Irimia et al. compared the headache frequency and quality of life measured by the Headache Needs Assessment (HANA) survey.^21^ This scale assesses current quality-of-life-related issues, such as lacking energy, and participation in social activities.^22^ The authors found a positive correlation between the number of monthly headache days and restraints in daily life imposed by the disorder—not a u-shaped correlation. However, as the HANA measures the *current* impact of the headache on life, and the SLS measures satisfaction with life in general, it is unclear how well the two scales correlate. Thus, no conclusion on the validity of our results can be drawn.

Many participants shared their advice on living a happy life despite their migraine attacks, suggesting that interference of migraine with happiness seems possible to them. Conversely, nobody mentioned any positive effects of bearing migraine attacks. Consequently, participants' replies also do not imply that headache *per se* leads to happiness—the interpretation mentioned above is more likely.

Interestingly, the strategies to increase happiness reported by the participants differed depending on disease duration. Those with a shorter duration often emphasized the importance of focusing on the days without pain and increasing their meaning. This finding suggests that these “beginner migraineurs” attempt to “counterbalance” the attacks by increasing and enjoying the pain-free time's positive aspects.

With increasing disease duration, migraineurs' attitudes changed as they increasingly focused their advice on the attacks themselves and reducing their impact. On the one hand, this finding might imply that migraine gets increasingly cumbersome with increasing duration. On the other hand, more experienced patients might simply have more advice to share on coping effectively with an attack. As disease duration had no impact on happiness, the latter option seems more likely.

Few migraineurs mentioned acute pharmacological treatment; nobody mentioned pharmacological prophylaxis in their answers to the free text question, despite one-third of the participants reporting taking one. The low adherence to preventive treatments documented in previous studies and our data suggest that pharmacological treatment options generally do not seem to occupy much space in patients' thoughts.^23^ Consequently, this may imply that patients intuitively assume great importance of lifestyle and mindset in the pathophysiology of new attacks.

### Strengths and limitations

To our knowledge, this is the first study investigating migraine and happiness. However, there are some limitations to be mentioned.

We did not validate participants' self-reported migraine diagnoses and the self-reported data. Even though we specified in each question whether it addressed migraine attacks or referred to headache attacks in general, we did not guide participants on how to distinguish them. Consequently, some participants might have conflated different headache types in their replies.

Furthermore, the sample was relatively small and—due to the sampling strategy—not representative. Also, we did not perform a power calculation before conducting the study as no preliminary data were available. Besides, we did not correct for multiple testing, as this study was exploratory, that is, we aimed to create new hypotheses instead of confirming them.

Finally, happiness could be defined and assessed differently. Examples are positive and negative affect,^3^ flourishing,^24,25^ and thriving.^26^ However, given the significant disease burden of migraine that comprises irreversible burden,^6^ we thought that “satisfaction with life” is an adequate measure of the impact of migraine on life.

## Conclusions

This study investigated the association between migraine attacks and happiness and found that most migraineurs were satisfied with their lives. However, surprisingly, a higher number of headache days were associated with more happiness.

More detailed analysis suggested that high numbers of headache days might be associated with less negative affective consequences of migraine attacks. Thus, the headache frequency alone does not necessarily correlate with well-being. The precondition for happiness seems to be constantly high or low exposure to negative influencing factors.

Overall, our data suggest that suffering from migraine attacks can nonlinearly complicate, but not entirely impede finding happiness.

## Abbreviations Used

GAD-7: Generalized Anxiety Disorder scale
MMD: monthly migraine days
n.r.: not reported
PHQ-8: Patient Health Questionnaire
SWLS: Satisfaction-With-Life-Scale
TLA: time lost due to an attack
